# The Results of Adductor Magnus Tenodesis in Adolescents with Recurrent Patellar Dislocation

**DOI:** 10.1155/2015/456858

**Published:** 2015-02-16

**Authors:** Krzysztof Malecki, Jaroslaw Fabis, Pawel Flont, Kryspin Ryszard Niedzielski

**Affiliations:** ^1^Clinic of Orthopaedics and Traumatology, Polish Mother's Memorial Hospital Research Institute, Rzgowska 281/289, 93-338 Lodz, Poland; ^2^Department of Arthroscopy, Minimally Invasive Surgery and Sports Traumatology, Medical University of Lodz, Żeromskiego 113, 90-549 Lodz, Poland

## Abstract

Recurrent dislocation of the patella is a common orthopaedic problem which occurs in about 44% of cases after first-time dislocation. In most cases of first-time patellar dislocation, the medial patellofemoral ligament (MPFL) becomes damaged. Between 2010 and 2012, 33 children and adolescents (39 knees) with recurrent patellar dislocation were treated with MPFL reconstruction using the adductor magnus tendon. The aim of our study is to assess the effectiveness of this surgical procedure. The outcomes were evaluated functionally (Lysholm knee scale, the Kujala Anterior Knee Pain Scale, and isokinetic examination) and radiographically (Caton index, sulcus angle, congruence angle, and patellofemoral angle). Four patients demonstrated redislocation with MPFL graft failure, despite the fact that patellar tracking was found to be normal before the injury, and the patients had not reported any symptoms. Statistically significant improvements in Lysholm and Kujala scales, in patellofemoral and congruence angle, were seen (*P* < 0.001). A statistically significant improvement in the peak torque of the quadriceps muscle and flexor was observed for 60°/sec and 180°/sec angular velocities (*P* = 0.01). Our results confirm the efficacy of MPFL reconstruction using the adductor magnus tendon in children and adolescents with recurrent patellar dislocation.

## 1. Introduction

Recurrent dislocation of the patella is a common orthopaedic problem which occurs in about 44% of cases after first-time dislocation [1]. Several dozen descriptions of surgical methods for recurrent patellar dislocation are given in the literature, and an appropriate one, such as proximal, distal realignment or sulcus plasty [2–5], must be selected depending on the severity of anatomical and functional problems, as well as the age of the patient. In most cases of first-time patellar dislocation, the medial patellofemoral ligament (MPFL), which is the most important stabiliser within the first 30 degrees of flexion, becomes damaged [6]. However, the MPFL may be reconstructed to realign a patella with deficient proximal medial restraints, a procedure which can be combined with lateral retinacular release, or a distal realignment technique such as tibial tuberosity osteotomy or patellar tendon partial transposition, in skeletal-immature patients experiencing patellar tilt or shift [2, 7–10].

An additional test rarely used in the diagnosis and treatment of recurrent patellar dislocation is based on isokinetic measurements. Isokinetic evaluation performed after surgery allows the rehabilitation programme to be optimised and is intended to return the muscle strength of the extensor system to a pretreatment status comparable to that of the healthy side. Testing the strength of the muscle is important because the vastus medialis oblique (VMO) serves to control patellar tracking through varying degrees of knee motion and prevents the occurrence of anterior knee pain [11]. In patellar instability, it is critical to maintain the dynamic balance of the quadriceps to limit the dominance of the lateral structures. Isokinetic testing evaluates the dynamic relationships during knee movements, and, as it is reproducible and fully objective, it may act as one of the endpoints in the study of patellar instability treatment methods [12–15].

## 2. Materials and Methods

Between 2010 and 2012, 33 patients (39 knees) with recurrent patellar dislocation were treated with MPFL reconstruction using the adductor magnus tendon. The average age at the time of surgery was 16 years (range: 8 to 18 years, SD 2.5). All patients (20 girls and 13 boys) were available for follow-up examination and were analysed prospectively. The mean follow-up was 2.6 years (range: 2 to 3 years, SD 0.5) (Table 1).

In the case of 9 knees with patellar shift and lateralisation of the tibial tuberosity, normal patellar tracking was restored by supplementing the MPFL reconstruction with the transfer of one half of the patellar tendon. In the case of 23 knees with patellar tilt, lateral retinacular release was performed. The following study inclusion criteria applied: patellar dislocation that had occurred at least twice, positive apprehension sign, completed research protocol, and age under 18 years at the time of surgery. Subjects with first-time patellar dislocation or habitual patellar dislocation, as well as patients with osteochondral fracture and those with a history of knee surgery, were excluded. Of the 39 knees which met the study inclusion criteria, 14 had experienced two dislocations, 16 three dislocations, and 9 multiple dislocations (from 4 to 12). The trochlea dysplasia and patella alta did not influence the treatment protocol.

The outcomes were evaluated functionally and radiographically. The number of recurrent dislocations was noted, and positive apprehension tests were carried out. Laxity analysis based on the Beighton scale was also performed: a score of ≥4 being considered as ligamentous laxity [16]. The evaluation also included patient-reported outcome measures (Lysholm knee scale and the Kujala Anterior Knee Pain Scale) and isokinetic examination [17, 18]. Quadriceps and hamstring maximal peak torque at 60 and 180°/sec were measured 1 month before and 1 year after surgery (Biodex Multi-Joint System-Pro, Biodex Medical Systems, Inc., New York, USA).

**Figure 1:**
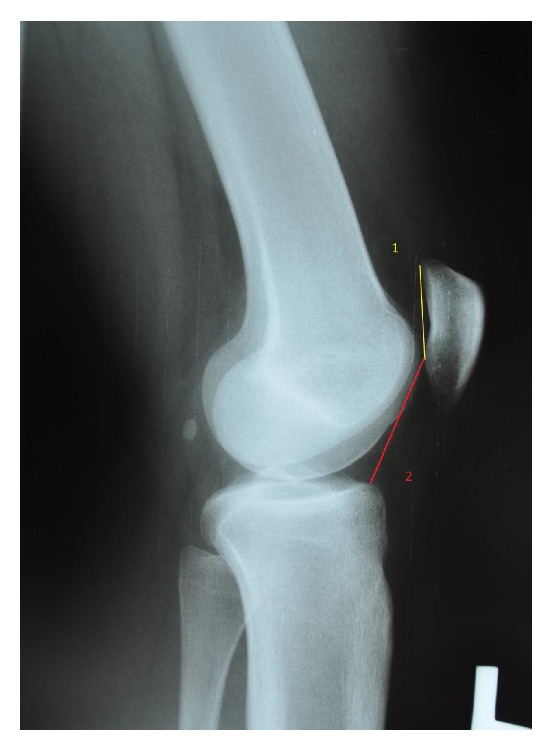
Example of Caton index measurement in lateral standing view. Caton index = L2/L1.

**Figure 2:**
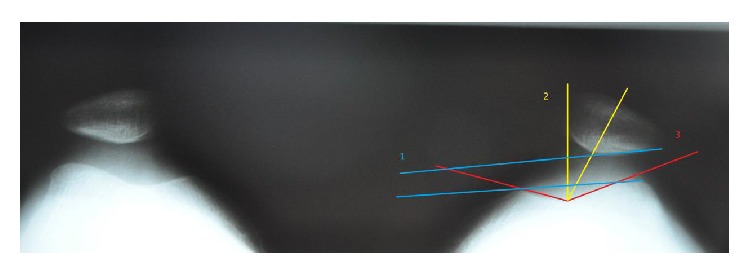
Preoperative X-rays according to Merchant view in 45° of flexion with quadriceps tension. (1) Patellofemoral angle. (2) Congruence angle. (3) Sulcus angle.

**Figure 3:**
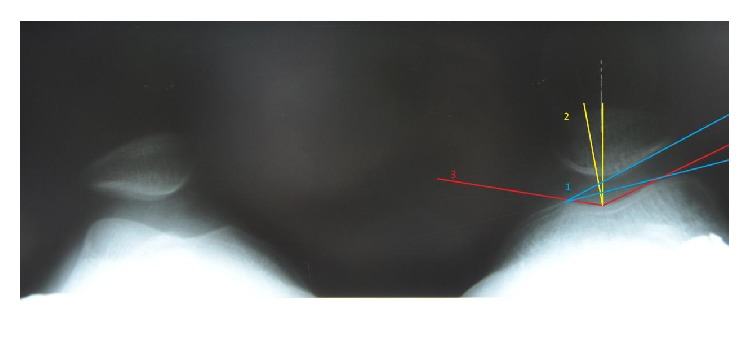
Postoperative X-rays according to Merchant view at 45° of flexion with quadriceps tension. (1) Patellofemoral angle. (2) Congruence angle. (3) Sulcus angle.

The radiological assessment before and 1 year after surgery included a lateral standing weight-bearing position and axial X-ray examination according to Merchant at 45 degrees of knee flexion. The following parameters were assessed: Caton index, sulcus angle, congruence angle, and patellofemoral angle. Patellar height was determined by lateral projection X-ray performed under full loading according to Caton (Figure 1), and sulcus angle, congruence angle, and patellofemoral angle were assessed based on axial radiograms obtained by Merchant view [4, 5, 14] (Figures 2 and 3). The following values were adopted as normal: sulcus angle ≤147°, patellar height ≤1.2, patellofemoral angle being open laterally (lower than 0°), and a congruence angle from −17° to 5°, with values greater than 5° implying patellar lateralisation [19–25].

## 3. Surgical Technique

**Figure 4:**
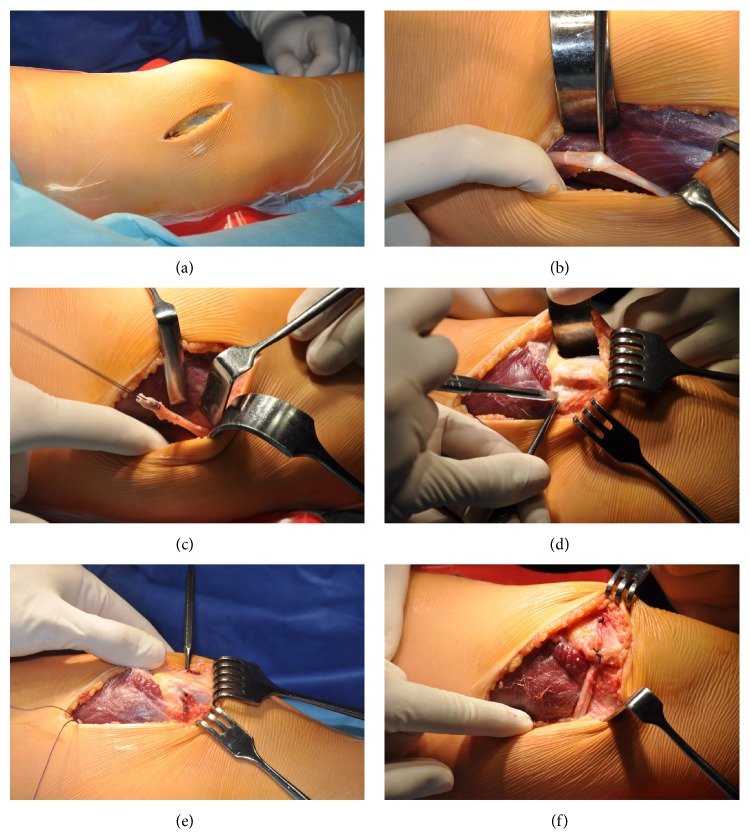
Surgical technique. (a) Incision. (b) Harvesting of the adductor magnus tendon lying under the vastus medialis. (c) Adductor magnus cut from its proximal insertion and suture with 1.0 Vicryl. (d) Preparation of the superomedial corner of the patellae. The knives show MPFL insertion to the patellae. (e) The patellae were drilled from the medial to lateral border. Drilling was started at the point where MPFL is inserted. (f) The adductor magnus tendon was passed through the patellae and sutured with 1.0 Vicryl with appropriate tension, with the knee flexed at 30°.

All patients were operated on by the first author according to the technique first described by Avikainen from 6 weeks to 12 weeks after last injury [26]. The aim of the rehabilitation program performed before surgery was to achieve full range of motion of the knee without effusion. A medial incision approximately 10–15 cm long was performed (Figure 4(a)). The deep fascia and retinacula were incised and the vastus medialis muscle was elevated anteriorly (Figure 4(b)), following which the adductor magnus tendon was dissected and cut from the musculotendinous junction, while the distal insertion was left intact (Figure 4(c)). A tunnel was created in the patella (Figures 4(d) and 4(e)), through which the tendon was sutured with Vicryl 1.0 at an appropriate tension with the knee flexed at 30° (Figure 4(f)). The graft was passed between the synovium and the fibrous membrane of the articular capsule. From the same incision, a lateral retinacular release was performed in the case of patellar tilt. Lateral release that was performed if the patellofemoral angle was found to be open medially in Merchant view X-rays and patellar tilt was present on physical examination. Tilt was assessed by palpating the medial and lateral borders of the patella with the knee extended or slightly flexed, as described by Kolowich et al. [27].

In nine of the knees, partial patellar medial transposition according to Roux-Goldthwait with resuturing under the periosteum medial to the tibial tuberosity was performed [21, 28]. Distal realignment was performed in the case of the Q angle being greater than 20° and the presence of a patellar shift with an increased congruence angle. According to Merchant, the normal value of congruence angle ranges from 5° to −17°. Orthosis immobilisation was used for 6 weeks. For the first 2 weeks, the orthosis was completely locked in a 10-degree flexion; for the next 2 weeks, a 0–30-degree range of motion was permitted in the orthosis, increasing to 0–60 degrees after another 2 weeks. The full loading of the operated limb was allowed 4 weeks after the procedure. Outpatient rehabilitation was carried out for 6 months.

The nonparametric Wilcoxon test for related data and Mann-Whitney *U* test for unrelated data were used to compare the frequencies of significant deficits, as was the independent *χ* ^2^ test for a four-field array. Statistical significance was assumed for *P* < 0.05. The analysis was performed using the STATISTICA software package, ver. 10 (Statsoft, Inc. 2011, DASS, http://www.statsoft.com/). The study was approved by our institutional review board (approval issue date October 23, 2010).

## 4. Results

Research protocol was completed in all patients. Recurrent dislocation during follow-up was reported in 4 patients during the 2- to 3-year period following surgery (4 knees, 10.3%): during sports in three cases and while dancing in one case. These patients experienced intra-articular haematoma following the recurrent dislocation, and ultrasound demonstrated signs of MPFL avulsion tear (*n* = 2) or rupture of the ligament (*n* = 2). The recurrent events occurred in patients without partial transposition of the patellar tendon.

While a positive apprehension test was seen in all patients at screening, it was positive only in 7 cases (17.9%) at follow-up; this number includes all patients with recurrent dislocation. All patients in the present study achieved the full range of knee motion and none reported excessive pressure of the patella. Twenty-one patients met the diagnostic criterion for ligamentous laxity (63.6%). The mean value in this scale for the entire cohort was 4.3 (range of 0 to 8). Statistically significant improvements were achieved according to both the Lysholm and Kujala scales (*P* < 0.001). The mean presurgery score was 64 points (ranging from 30 to 95 points), increasing to 91 points at follow-up (59 to 100 points) according to the Lysholm scale, while the mean presurgery score was 66 points (38 to 88 points) increasing to 92 points at follow-up (70 to 100 points) according to the Kujala scale (Table 1).

## 5. Imaging

Statistically significant improvements in patellofemoral and congruence angle were seen (*P* < 0.001). Patellar tilt was observed in 25 knees preoperatively and in 7 after surgery, whereas patellar shift was found in 32 knees preoperatively and in 10 knees after surgery. No statistically significant improvement was observed in sulcus angle (*P* = 0.07) or Caton index (*P* = 0.614), and no improvement was found in any of the 28 knees with abnormal sulcus angle. Similarly, while the Caton index was normalised in 7 knees, it remained abnormal in 16 knees (Table 2).

## 6. Isokinetic Evaluation

A statistically significant improvement in the peak torque of the quadriceps muscle was observed for both tested angular velocities (*P* = 0.01). Improvement was seen in 24 operated limbs when measured at a speed of 60°/sec and in 26 operated limbs at 180°/sec. A deficit of peak torque of more than 10% relative to the opposite limb after surgery was found in 24 limbs when tested at 60°/sec and in 20 limbs at 180°/sec.

As with the quadriceps examination, the isokinetic evaluation of the flexor revealed a statistically significant increase of peak torque for both angular velocities after treatment (*P* < 0.001). Progress was noted in 30 knees at 60 degrees/sec and in 34 knees at a speed of 180°/sec. A peak torque deficit of more than 10% relative to the opposite limb in the flexors after surgery was found in 10 limbs when tested at 60°/sec and in 14 limbs at 180°/sec (Table 3).

## 7. Discussion

The most important finding in the present study was that the described surgical technique is efficacious in prevention of recurrent patellar dislocation. Four patients (10.3%) demonstrated recurrence with MPFL graft failure, despite the fact that patellar tracking was found to be normal before the injury causing redislocation, and the patients had not reported any symptoms.

A statistically significant improvement in patient-reported outcome according to the Lysholm and Kujala scale was observed throughout the entire cohort, which confirms the effectiveness of MPFL reconstruction. These findings are supported by the results of the radiological examination, which showed improvement in the two most important indicators of the patellar position: the congruence angle and the patellofemoral angle.

Avikainen et al. observed a recurrent dislocation in 1 of 14 patients examined in a 7-year follow-up period after adductor magnus tenodesis; however, the inclusion criteria were different than those used in the present study, because first-time dislocations were included [26]. Another study by Sillanpää et al. compares the effectiveness of MPFL reconstruction with that of distal realignment in treating recurrent patellar dislocation [29]. The study group included 15 patients with MPFL reconstruction from the tendon of the adductor magnus muscle, and 21 patients after a Roux-Goldthwait operation. The MPFL group included 10 good or very good outcomes, 5 satisfactory and poor outcomes, and 1 patient with a recurrent dislocation. Similarly, in the Roux-Goldthwait group, there were 12 good and very good outcomes, 9 poor and satisfactory outcomes, and 3 cases of recurrent dislocation. Follow-up X-ray scans revealed no degenerative lesions in the MPFL group but 5 cases in the Roux-Goldthwait group.

Panagopoulos describes a technique of MPFL reconstruction using a semitendinosus tendon which is passed through the medial intermuscular septum at the adductor magnus insertion. This graft, where the intermuscular septum acts as a pulley for the tendon, functions similar to the reconstructed MPFL used in our study. In short follow-up of 25 patients, no cases of redislocation were recorded and one patient sustained a patellar facture. The author reports improvement in IKDC, Tegner, Lysholm, and Kujala scores [30].

In 2009, Sillanpää et al. published a minimally invasive method of tendon collection, which resulted in a reduction in wound length [31]. However, in the authors' experience, due to the vascular topography of anatomical variants of vessels, the preferred method for tendon harvest should be the open method. It allows simultaneous lateral retinacular release from the same incision if necessary. If arthroscopy is performed before MPFL reconstruction, lateral retinaculum release is carried out with a coagulation device which is placed in the inferolateral portal. Arthroscopy is also useful for diagnosis and treatment of the cartilage lesion at the medial patellar facet and the lateral edge of the femoral trochlea.

Although a comprehensive literature review reveals no other descriptions of this specific surgical procedure, many other methods for MPFL tendon reconstruction with free grafts from the gracilis, semitendinosus, quadriceps tendon, fascia lata, or iliotibial band have been reported. The recurrences of dislocation observed with various grafts are similar and range from 5 to 10% [32–38].

The findings of the present study do not reveal any improvements of patellar height ratio or sulcus angle after surgery. In principle, no such changes were to be expected with this method, as trochlear rebuilding is only theoretically possible in young patients with long-term follow-up, and it should not be expected in the adolescent group [39, 40]. Another possible concern is based on the nonanatomical location of the graft and its adverse impact on the change in patellar height; however, as the average Caton index was found to decrease slightly, no such relationship was confirmed; as a result, the indicator was found to be normalised in more patients at the follow-up assessment. The femoral insertion on the adductor tubercle is very close to the native femoral MPFL attachment and in the anatomic study ranges from 6 mm to 15 mm, mean 11 mm [41]. In different cadaveric study the medial patellofemoral ligament attachment on the femur was 1.9 mm anterior and 3.8 mm distal to the adductor tubercle [42].

The isokinetic analysis showed a significant improvement in peak torque of the quadriceps and flexors for both speeds evaluated. It should be noted, however, that a clinically significant deficit of greater than 10% remained, compared to the opposite side, which may lead to an increased risk of injury [14]. Treatment and rehabilitation after surgery significantly decreased the number of deficits in relation to the opposite side only in the flexor examination at 60 degrees/s. Hence, there is a need for a comparison of isokinetic testing between the two sides to be incorporated in any planned rehabilitation programme aimed at regaining extensor muscle strength.

A prospective study of 20 patients evaluated before and after a procedure for recurrent patellar dislocation based on isokinetic evaluation at speeds of 6, 12, and 60°/sec was published by Rauschning et al. In a 20-month follow-up period, the authors reported a slight, nonsignificant improvement in the torque values of the quadriceps muscle, as well as a significant decrease in performance compared with healthy volunteers from a control group [43]. Similar to the present study, Ronga et al. report persistent significant weakness in the operated limbs of 28 patients with recurrent patellar dislocation, compared to the opposite limbs, at the last follow-up assessment, with a mean follow-up of 3.1 years [12]. Similarly, in a prospective study involving 25 sport-practising skeletally immature patients with recurrent patellar dislocation, Oliva et al. reported a decrease in the muscular parameters in the operated limbs after an average period after surgery of 3.8 years [13].

The procedure described by Avikainen allows autologous, vascularised graft to be used in growing patients with open growth cartilages. As most other methods of MPFL reconstruction involve the risk of damage to the growth cartilage when fixing the graft, these procedures are mainly performed in adults. However, the perceived disadvantages of this method include the slightly nonanatomical location of the graft and the risk of damage to the femoral vessels, the saphenous branch of the descending genicular artery and the saphenous nerve which lie close by [41, 42]. An analysis of surgical material indicates the most significant limitations of this method to be the lack of tendon in some cases and its insufficient length or small cross section, all of which make reconstruction impossible. In the present study, no tendon was found intraoperatively in two knees, and the graft was too short in two others; these patients were not included in the study.

## 8. Conclusions

Our results confirm the efficacy of MPFL reconstruction using the adductor magnus tendon in children and adolescents with recurrent patellar dislocation. In the presence of a deficit in quadriceps and flexor strength, patients may benefit from isokinetic analysis when planning the postoperative rehabilitation programme following MPFL reconstruction with an adductor magnus tendon.

## Figures and Tables

**Table 1:** Demographic data and clinical outcomes according to the Lysholm, Kujala, and Beighton scales.

	*X*	Range	SD	Me
Age at the time of surgery (years)	16	8–18	2.7	17
Follow-up (years)	2.6	2-3	0.5	3
Lysholm scale pre-/postoperation	64/91	30–95/59–100	14/11	62/95
Kujala scale pre-/postoperation	66/92	38–80/70–100	11/9	68/95
Beighton scale	4.3	0–8	2.1	4

**Table 2:** Radiological outcomes in screening and check-up.

	*X* Me		SD range		Normal values (*n*)		Abnormal values (*n*)		*P* value
	Pre	Post	Pre	Post	Pre	Post	Pre	Post	
Sulcus angle [°]	149 148	148 150	6.8 135–164	7.1 130–160	11	11	28	28	*P* = 0.801
Caton index	1.27 1.28	1.24 1.14	0.26 0.77–2	0.3 0.93–2	16	23	23	16	*P* = 0.117
Patellofemoral angle [°]	4 2	−4 −5	10 −10–34	5 −15–8	14	32	25	7	*P* < 0.001
Congruence angle [°]	21 17	−5 −2	20 −15–75	12 −30–20	7	29	32	10	*P* < 0.001

**Table 3:** Data for isokinetic measurement during screening and check-up.

		Progress of peak torque (*n* = 39)		Peak torque deficit >10% (*n* = 39)		
		No	*P* value	Screening	check-up	*P* value
Maximal quadriceps muscle torque values	At 60°/s	24	*P* = 0.010	19	24	*P* = 0.255
	At 180°/s	26	*P* = 0.010	16	20	*P* = 0.346

Maximal knee flexor muscle torque values	At 60°/s	30	*P* = 0.001	15	10	*P* = 0.027
	At 180°/s	34	*P* = 0.001	13	14	*P* = 0.811
